# Abundance of Inflammatory Response Genes Among Cardiovascular Disease and Ischemic Stroke Genes

**DOI:** 10.3390/ijms27104442

**Published:** 2026-05-15

**Authors:** Gennady V. Khvorykh, Ivan B. Filippenkov, Andrey V. Khrunin, Lyudmila V. Dergunova, Svetlana A. Limborska

**Affiliations:** Laboratory of Human Molecular Genetics, National Research Centre “Kurchatov Institute”, Kurchatov Sq. 1, Moscow 123182, Russia; gennady.khvorykh@gmail.com (G.V.K.); filippenkov-ib.img@yandex.ru (I.B.F.); khrunin-img@yandex.ru (A.V.K.); dergunova-lv.img@yandex.ru (L.V.D.)

**Keywords:** inflammation, ischemic stroke, cardiovascular disease, human genes, rat gene orthologues, tMCAO, gene expression

## Abstract

Inflammation plays a key role in the pathogenesis of many diseases, including cardiovascular disease and ischemic stroke. However, despite the existence of known inflammatory genes, the question of estimating their total number and the possibility of discovering new ones remains open. This study sought and analyzed genes involved in inflammation among genes related to cardiovascular disease and ischemic stroke. Human genes associated with ischemic stroke (N = 1177) and cardiovascular disease (N = 1756) were retrieved from the DisGeNET platform. Inflammatory and immune response genes were obtained from the Gene Ontology, NCBI, and Reactome databases. An additional list of 140 inflammatory genes was compiled based on our previously obtained data on the differential gene expression in a rat brain under transient middle cerebral artery occlusion. Genes that occurred simultaneously in both the inflammatory gene lists and gene lists of diseases were selected and considered. The resulting combined gene list included 1285 inflammatory genes. The *NFKB1* and *RELA* genes demonstrated the highest frequencies across the various inflammatory gene selection resources we examined. Using a combination of experimental and bioinformatics approaches, a representative list of inflammatory genes important for the pathogenesis of ischemic stroke was compiled. The identified genes may be crucial for the development of anti-inflammatory therapeutic strategies for this disease.

## 1. Introduction

The role of inflammation in cardiovascular diseases (CVDs) is actively investigated both before and after their clinical manifestation. Cerebral ischemia triggers an acute inflammatory response to necrotic cell and tissue death resulting from oxygen deprivation [1]. However, the role of the immune system in the development and progression of CVDs and IS is not always straightforward and remains insufficiently understood. For instance, associations have been demonstrated between immune cell phenotypes and susceptibility to stroke [2], which could be critical for developing stroke prevention strategies and predicting its outcomes, including approaches based on the analysis of individual inflammatory gene profiles [3,4]. Another reason uniting CVD and ischemic stroke is atherosclerosis. Despite the presence of various genetic markers of CVD and ischemic stroke, the relationship between coronary and carotid artery disease is well recognized [5]. It is the common risk factor, the pathophysiological basis of which is a chronic inflammatory process caused by endothelial damage and lipid metabolism disorders, leading to the formation of atherosclerotic plaques [1]. This systemic disease affects arteries throughout the body, including the heart and brain. The clear relationship between coronary and carotid artery disease allows us to consider CVD and ischemic stroke together in the context of their pathogenesis in the hopes of identifying common biomarkers for the early prognosis of each disease [6].

This study aimed to outline a general pool of inflammatory genes associated with CVDs and/or IS genes. Since the most therapeutically promising inflammatory targets are likely those that operate across different phases of diseases, we included genes linked to both predisposition and severity, capturing the full spectrum of inflammation’s role across the entire disease continuum—from susceptibility to outcome. The core logic was that the inflammatory response is not a static event but a dynamic process that contributes to disease initiation, progression, and health recovery. This continuum suggests that key inflammatory pathways may be operative across the entire disease spectrum, from susceptibility to severity and outcome, a view supported by integrative models of stroke pathophysiology [7].

In this study, we considered both standard databases and the specialized sources of genes related to inflammation. Specifically, we included in this analysis a list of inflammatory genes compiled from our prior data on the expression of their orthologues in a rat model of cerebral ischemia–reperfusion (transient middle cerebral artery occlusion, tMCAO), since our previous cross-species studies demonstrated that differentially expressed genes (DEGs) in the rat brain under experimental cerebral ischemia serve as an effective source of candidate genes for investigating the genetic determinants of IS in humans, and these stroke-associated genes were specifically implicated in inflammatory processes [8].

Through a combined analysis of gene lists related to inflammation, CVDs, and/or IS, we have established representative lists of inflammatory genes that may be important for the biological processes underlying CVDs and/or IS and hold potential for the development of therapeutic strategies.

## 2. Results

Our research focused on the global genetic landscape of IS, CVDs related to the genes of inflammation and the immune system. To conduct the study, we considered 11 lists of genes related to inflammation and/or the immune system. Eight lists (1st–8th) related to inflammation and the immune system were represented by the genes from GSEA and NCBI databases, and the genes that were specifically collected for conducting genetic association studies (2007_Assembly). Two lists of genes associated with IS and CVDs (disease genes) were retrieved from the aggregator base DisGeNET. Additionally, one list of inflammation genes was identified among the human orthologues of DEGs of rats under experimental ischemia (Table 1).

**Figure 1:**
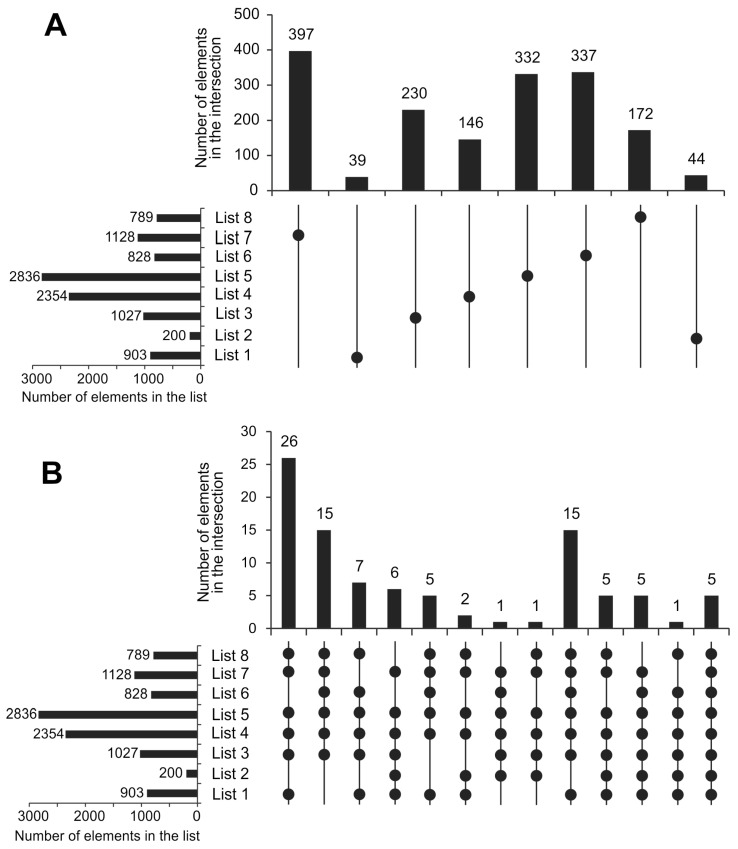
The Upset diagrams of inflammatory genes analyzed that are unique to each list (**A**) or found in six or more lists (**B**), with the search attributes given in Table 1. The number of gene lists corresponds to those given in Table 1. The horizontal bars represent the number of genes in each list. In the matrix, a filled dot indicates that a set is included in the intersection (AND). Empty spaces indicate that a set is not included in that specific intersection (NOT). Connected dots show that multiple sets intersect simultaneously. The vertical bars indicate how many genes belong specifically to that intersection.

The total pool of inflammatory genes comprises 4585 unique ones. The number of unique inflammatory genes and those found in six or more lists of inflammatory gene lists are given in Figure 1A and Figure 1B, respectively. Among these, 1697 genes were found in only a single database, while 2889 genes were present in two or more databases. The presence of both shared and unique genes in the inflammatory gene lists indicates the utility of the compiled data for ranking disease genes based on their frequency across the discussed lists and for revealing candidate genes associated with diseases, as described below.

To define the pool of genes associated with CVDs and IS, the DisGeNET database was used. The DisGeNET database accumulates the genes associated with disease, disregarding the stages of disease, molecular levels of investigation, and species. We retrieved 1177 and 1756 genes associated with IS and CVDs, respectively, from DisGeNET.

The total number of disease genes was 2315. Among these, 618 genes overlapped between genes annotated for CVDs and those specifically for IS, characterizing the spectrum of IS genes as a substantial component of CVDs. Fisher’s exact test showed that the observed overlap between the disease genes and each of the inflammatory gene lists exceeds expectation under a null model of random sampling from the human protein-coding genome (N = 19,500). For all 16 intersections of pairs of gene lists, the enrichment was highly significant (*p* < 0.0001) (Table S2).

We cross-referenced the obtained gene lists by calculating how frequently the disease genes appeared in the inflammatory gene lists. In total, 1219 such genes associated with IS or CVDs were identified. The genes *NFKB1* and *RELA* were the most frequently represented across inflammatory gene lists, showing the highest occurrence in 8 lists annotated for “inflammation”. The list of inflammatory genes also included *NFKBIA*, *RIPK2*, *CYBB*, *HMGB1*, *IKBKB*, *IL1B*, *ITGB2*, *PTAFR*, *CCL22*, *SYK*, *TLR1*, *TLR2*, *TNFRSF1B*, *CD14*, *CD36*, and *CD40*. The results of the intersections of all lists of genes considered are given in the form of a binary matrix in Table S3.

The resulting gene list was supplemented with genes identified as DEGs in the experimental model of cerebral ischemia (tMCAO). We found 143 rat genes meeting the described above search criteria (Table S1). Human orthologs were identified for 140 of them. Interestingly, we detected 51 DEGs at 4.5 h after tMCAO in rats (Table S1). Among them were *Cd14*, *RT1-Da*, *RT1-Db1*, *Casp4*, *Ccl2*, *Tnfrsf1a*, *Prkcd*, *Anxa1* and others. All of them were also found among the DEGs at 24 h after tMCAO. These genes were mainly associated with the AGE-RAGE signaling pathway in diabetic complications, inflammatory bowel disease, Toll-like receptor, and other signaling pathways, according to DAVID data. Then, 92 genes (e.g., *Casp1*, *Ccl7*, *Pla2g4a*, *Gsdmd*, *S100a8*, *Txn1*, *S100a9*) were specific for the 24 h time point after tMCAO in rats (Table S1). These genes were associated mainly with the inflammation mediator regulation of TRP channels, the GnRH signaling pathway, lipid and atherosclerosis, and other pathways, according to DAVID data.

**Figure 2:**
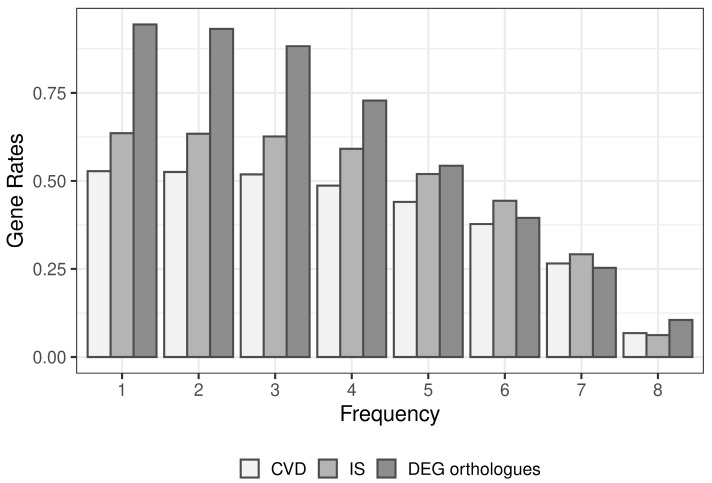
The rates of disease genes met at least 1 to 8 times in the lists of inflammatory genes from the main pool. CVD—cardiovascular disease, IS—ischemic stroke, DEG orthologues—human orthologues of DEGs of rat under tMCAO.

Adding these genes to the list of inflammatory genes found to be associated with IS or CVDs from the database analysis brought the total number of inflammatory genes to 1285 (Table S3). Table S4 listed the 24 genes (*CCL2*, *CCL5*, *CD14*, *CD36*, *CD40*, *NFKBIA*, *RELA*, *S100A9*, *TNFRSF1B*, *CASP1*, *HMGB1*, *ITGB2*, *IL1A*, *IL1B*, *IL18*, *IL4R*, *IL6*, *MAPK8*, *NFKB1*, *PIK3CD*, *PRKCD*, *RIPK2*, *TLR2*, *TLR4*) presented in analyzed resources ≥9 times and with inflammatory functions and disease associations. In addition to ischemia, among the diseases found were Liver Cirrhosis, Schizophrenia, Pneumonia, Allergic Reaction, and others. The cumulative proportions of disease genes with varying frequencies of occurrence in the inflammatory gene lists are presented in Figure 2. It was found that 53% of CVD genes, 64% of IS genes, and 95% of human orthologues of the DEGs in the rat tMCAO model appeared at least once in the inflammatory gene list. To further check the biological relevance, the functional enrichment analysis (Gene Ontology (GO), KEGG, Reactome pathways) for the final 1285 genes was carried out (Table S5). There were 198 KEGG and 454 Reactome pathways, as well as 214 GO molecular function annotations with padj < 0.05. Among the most significant pathways were the Immune System, Cytokine Signaling in the Immune System, Signaling by Interleukins, Lipid and atherosclerosis, Inflammatory mediator regulation of TRP channels, and others. In addition, genes were related to molecular functions, such as signaling receptor binding, identical protein binding, cytokine activity, enzyme binding, and others. Thus, the impact of inflammatory pathways was highlighted.

To assess the clinical relevance of the combined 1285 inflammatory genes, we queried the NHGRI-EBI GWAS Catalog. A total of 1024 genes (80%) were present in the catalog (Table S6). The associated traits included ischemic stroke, cardiovascular disease, and a broad spectrum of related risk factors such as hypertension, lipid levels, body mass index, and others. This high proportion indicates that our inflammation-focused gene set is substantially enriched for disease-related loci with documented human genetic variation. The remaining 261 genes (20%) were not found in the GWAS Catalog, which may reflect their potential roles in less commonly examined phenotypes, or, in some cases, species-specific responses identified in animal models, particularly in the rat tMCAO model.

The genes *NFKB1* and *RELA* showed the maximum occurrence (11) across all the investigated resources used for selecting inflammatory genes. Fifteen genes (*ASIC4*, *CALM2*, *CALML4*, *HTR2B*, *MAF*, *NLRP1A*, *PRKCG*, *RT1-BA*, *RT1-BB*, *RT1-DA*, *RT1-DB1*, *TXN1*) were identified solely as orthologues of DEGs and were not defined as attributes of inflammation or involvement in CVDs or IS. Furthermore, 59 orthologues of post-tMCAO DEGs (*MYD88*, *PLCG1*, *TLR6*, *IFNGR2*, *NFKB2*, *PLCG2*, *PIK3R2*, *PYCARD*, *IFNGR1*, *IL1RAP*, *CCL4*, *GSDMD*, etc.) were found among inflammatory genes but not among CVD or IS genes. Based on the tMCAO data, two additional genes (*ADCY9*, *HTR2C*) were identified by us as inflammatory genes among the genes linked to CVDs or IS, which may reflect the specificity of the model animal.

A comparison of disease gene lists that occurred most frequently (in 7 or more resources) among inflammatory genes also revealed several genes specific to ischemic stroke (*IKBKB* and *SYK*), cardiovascular diseases (*NFKBIA*, *RIPK2*, *CYBB*, *PTAFR*, *CCL22*, and *TLR1*), and human orthologues of rat DEGs (*MYD88* and *PLCG1*). This finding suggests the specificity of molecular signaling in different pathologies, as well as its particular features in model animals.

## 3. Discussion

Inflammation is a primary driver of both IS and CVDs, with the immune system orchestrating the entire process. Analyzing jointly the genes related to IS, CVDs, inflammation, and immune response allowed us to identify that inflammation is a major shared mechanism underlying both conditions.

Our analysis included a total of 4585 inflammation-related genes, characterized by a substantial core of 2889 genes shared across multiple repositories. This high degree of overlap across eight independent sources provides a robust, cross-validated foundation for our inflammatory signature. However, the identification of 1697 genes unique to individual databases underscores the necessity of our integrated multi-source approach. Relying on a single repository would result in an incomplete ‘inflammatome,’ missing specialized genetic associations and emerging functional annotations. By synthesizing canonical pathways with disease-specific association lists, we captured a more comprehensive landscape of the inflammatory response than is possible through standard single-database queries.

In our study, we used an integrative approach combining the analysis of available sources from databases, as well as our own transcriptomic data under the conditions of the rat ischemic stroke model (tMCAO). It is important to note that all data were obtained in our laboratory under experimental conditions as close as possible and with maximum information about variations in these conditions, which minimizes the chance of artifacts and false positives. We consider this a competitive advantage, as the study could have been methodologically based on the use of disparate data sets published by various research groups. As a result, bioinformatic analysis of such data often encounters problems with batch effects, uncontrolled sample effects, and experimental conditions, which fundamentally reduce the reliability and quality of the results. Undoubtedly, there are limitations to organism-level models. Models based on rats are the most commonly used. Therefore, the rat-human orthologue approach is the most logical. There are also pig models [10,11,12]. These models have practical value for translational medicine due to the convoluted (folded) structure of the brain, a similar white-to-gray matter ratio, and comparable vascular anatomy. However, they are more difficult to implement.

We showed that well-established inflammatory genes (e.g., *TNF*, *IL6*, *CRP*) were present in the final inflammatory gene list. This result demonstrates the validity of our study. Furthermore, we were able to find inflammatory genes that were not found among the inflammatory genes for IS or CVD using the databases. Especially, using the results based on tMCAO, *ASIC4*, *CALM2*, *CALML4*, *MAF*, and *PRKCG* were found. In addition, a number of genes: *Cd14*, *RT1-Da*, *RT1-Db1*, *Casp4*, *Ccl2*, *Tnfrsf1a*, *Prkcd*, *Anxa1* were associated with inflammation and were differentially expressed already after 4.5 h after tMCAO, whereas *Casp1*, *Ccl7*, *Pla2g4a*, *Gsdmd*, *S100a8*, *Txn1*, *S100a9*, and other genes were specific DEGs for the 24 h time point after tMCAO in rats. Human orthologs of these genes can reflect early or later time points of different inflammatory phases.

The highest occurrence among the sources of inflammation and disease genes considered was demonstrated by the *NFKB1* and *RELA* genes. They presented in eight lists of inflammation genes, in CVD and IS genes, as well as in the human orthologues of DEGs of rats under tMCAO. The protein products of the *NFKB1* and *RELA* genes form the NF-κB heterodimer, a key transcription factor for numerous genes of innate and acquired immunity [13]. Upon receiving signals from cell receptors, this factor is activated, translocated to the nucleus, and initiates the transcription of genes that alter cellular function. Thus, NF-κB is a rapid-response factor to cellular stimuli, which is consistent with our observation of *NFKB1* and *RELA* present across all inflammatory gene lists for the considered diseases. The *NFKBIA*, *RIPK2*, *CYBB*, *HMGB1*, *IKBKB*, *IL1B*, *ITGB2*, *PTAFR*, *CCL22*, *SYK*, *TLR1*, *TLR2*, *TNFRSF1B*, *CD14*, *CD36*, and *CD40* were next more frequent inflammatory genes. They encode cytokines and their receptors, transcription factors, proteins responsible for antigen-presenting cell stimuli, cytokine mediators, high-mobility group proteins, and others [14,15].

A methodological note is warranted regarding the high frequency of *NFKB1* and *RELA* across Lists 1–8. Because these lists are not completely independent—they derive from interconnected ontologies and shared literature—the observed frequency may be inflated for most well-studied “canonical” inflammatory genes. Nevertheless, the independent confirmation of these genes in the disease-specific DisGeNET lists (CVD and IS) and in the tMCAO-derived orthologue list supports their genuine centrality to inflammation in cerebrovascular and cardiovascular pathology. Thus, while absolute frequency should not be interpreted as a statistically corrected measure, the consistency of annotation across multiple, partially overlapping resources remains biologically informative.

The 80% overlap between our inflammatory gene list (N = 1285) and the GWAS Catalog supports the translational relevance of our approach. However, the absence of 20% of genes from GWAS should not be interpreted as a lack of clinical importance. GWASs are inherently biased toward common variants associated with disease risk, whereas inflammation contributes to multiple stages of disease pathogenesis (i.e., initiation, progression, and resolution), which are not well captured by standard case–control designs.

This seems to be supported by a literature review of the noted inflammatory genes in the context of their involvement in IS pathogenesis, which revealed that associations of these genes with disease severity were more frequently reported than associations with disease risk [16,17,18,19,20]. This may be due to the differences between acute and chronic inflammation, respectively. The former occurs intensively, with maximal involvement of receptor and signaling systems and molecules, accompanied by the rapid development of clinical symptoms and outcomes. The latter develops over a long period without the manifestation of extreme levels of key inflammatory molecules [13], thereby complicating association studies.

A methodological limitation of this study concerns the use of DisGeNET for retrieving disease-associated genes. As a literature-derived knowledgebase, DisGeNET is subject to ascertainment bias, wherein well-studied genes (e.g., *NFKB1*, *RELA*, *IL1B*) are overrepresented due to higher publication volume, while under-investigated genes may be absent despite genuine biological relevance.

However, our study design incorporates several complementary strategies to mitigate this limitation. First, we integrated disease gene lists from DisGeNET with eight independently curated inflammation-related resources (GO, NCBI, Reactome, GSEA), each derived from different annotation methodologies and literature corpora. Second, we included experimental transcriptomic data from a rat tMCAO model, which is unbiased by prior human genetic literature and captures the gene-associated inflammatory responses that are not fully represented in DisGeNET. Third, as an independent validation, we cross-referenced our final gene set with the NHGRI-EBI GWAS Catalog and found that 80% (1024/1285) of genes had documented GWAS associations, indicating that our findings are not merely an artifact of DisGeNET’s biases. Nevertheless, we acknowledge that the 261 genes absent from GWAS—particularly the 59 orthologues identified exclusively from the rat model—may include both genuinely novel candidates and false positives requiring further functional validation. Future studies utilizing alternative bioinformatic and experimental approaches will be necessary to fully assess the clinical relevance of these genes.

## 4. Materials and Methods

For the purpose of this study, a gene was considered “inflammatory” if it was involved in the body’s immune system-mediated response to cellular injury. Thus, the corresponding genes were retrieved from databases using the queries containing the words “inflammatory,” “inflammation,” “inflammatory response,” and “immune system” (Table 1). The specific architecture of databases is shown in Table S7.

We acknowledge that Lists 1–8 are not statistically independent; therefore, frequency counts across these lists are used descriptively to assess annotation consistency, not as a probabilistic measure. Biological importance was further evaluated using independent methods (GWAS and functional enrichment).

Gene lists associated with the diseases under consideration were retrieved from the DisGeNET database version 2020 (https://disgenet.com, accessed 14 April 2024) [21]. The genes related to CVDs and IS were mainly retrieved with the keywords “cardiovascular disease” and “ischemic stroke”, respectively. In the case of IS, additional keywords were also applied. The sources, search attributes, and the number of genes obtained and considered are given in Table 1.

Then, we selected the DEGs in rats’ brains under tMCAO relative to sham-operated animals at 4.5 and 24 h after occlusion, identified with raw fold change ratio > 1.5 and padj < 0.05, where padj is the *p*-value adjusted with the Benjamini–Hochberg procedure. The respective transcriptomes were obtained and analyzed by us previously and are presented in Table S1.

Enrichment of disease-associated genes (CVD, IS) within the inflammatory gene lists was assessed using one-tailed Fisher’s exact tests. The background gene set consisted of all protein-coding human genes annotated in Ensembl GRCh38 (N = 19,500). Expected counts were calculated as (size of query gene set × size of inflammatory gene set)/background size. All analyses were performed in R version 4.5.3. *p*-values < 0.05 were considered statistically significant.

We compared these DEGs with the lists of genes presented in the KEGG Pathway, Reactome Pathway, Wikipathways, Gene Ontology, and Functional Category modules of the online service DAVID, 2021 Update, and v. 6.8 [22]. The final list included DEGs that were found in at least one of the mentioned databases and whose annotation attributes had the keyword “inflam$”. To validate the relevance of the final inflammatory gene list, DAVID (2021 Update) [22] was used to annotate the gene functions (GO, KEGG, Reactome pathways). Only functional categories with padj < 0.05 were taken into consideration. The DisGeNET, GAD_Disease, and GAD_Disease_Class databases were used to annotate gene-disease associations using DAVID.

The human orthologs of DEGs of rats were identified with Gene and Ortholog Location Finder of the Rat Genome Database (https://rgd.mcw.edu) and with the Alliance of Genome Resources platform (https://www.alliancegenome.org).

To assess the clinical relevance of the identified inflammatory genes, we also queried the NHGRI-EBI GWAS Catalog (https://www.ebi.ac.uk/gwas, accessed 16 April 2026) using the “REPORTED GENE(S)” field. Genes present in at least one GWAS association report were considered as having documented genetic association evidence.

Genomic coordinates of human genes under the genome assembly GRCh38 were retrieved from the Ensembl Genes 113 database via the Biomart service of the Ensembl project (https://www.ensembl.org). Data processing, analysis, and visualization were performed using custom scripts written in the R programming environment (version 4.5.3). The R packages UpSetR (version 1.4.0) and ggplot2 (version 4.0.1) were applied to visualize the data.

## 5. Conclusions

Our analysis of genes described as inflammatory and CVD- and IS-associated genes, combined with inflammatory genes whose orthologues exhibited altered expression in a rat tMCAO model, yielded a final list of 1285 inflammatory genes. It was shown that inflammatory genes constitute no less than half of all genes associated with CVDs and IS. Thus, by employing a combination of experimental and bioinformatic approaches, we have established a representative list of inflammatory genes that are important for the development of CVDs and IS and may have potential value for the development of anti-inflammatory treatment strategies.

## Figures and Tables

**Table 1:** The list of human genes considered in the research.

List	Search Attributes	Number of Genes	Source
List 1	GOBP_INFLAMMATORY_RESPONSE	903	gsea-msigdb.org
List 2	HALLMARK_INFLAMMATORY_RESPONSE	200	gsea-msigdb.org
List 3	The panel of inflammation-related genes for microarray (2007_Assembly)	1027	[9]
List 4	“Inflammatory genes” AND “Homo sapiens” (NCBI_inflammatory)	2354	ncbi.nlm.nih.gov
List 5	“Inflammatory response” AND “Homo sapiens” (NCBI_inflammatory_response)	2836	ncbi.nlm.nih.gov
List 6	REACTOME_ADAPTIVE_IMMUNE_SYSTEM	828	gsea-msigdb.org
List 7	REACTOME_INNATE_IMMUNE_SYSTEM	1128	gsea-msigdb.org
List 8	REACTOME_CYTOKINE_SIGNALING	789	gsea-msigdb.org
List 9	«Cardiovascular Disease» (cardiovascular_diseases)	1756	disgenet.com
List 10	«Ischemic stroke», «thromboembolic stroke», «cardioembolic stroke», «stroke, ischemic, susceptibility to», «cerebellar stroke», «stroke, lacunar», «embolic stroke of undetermined source», «stroke of undetermined etiology» (ischemic_stroke)	1177	disgenet.com
List 11	Human orthologues of DEGs of rats under tMCAO (ischemic_stroke_orthologues)	140	Table S1

## Data Availability

The original contributions presented in this study are included in the article and Supplementary Materials. Further inquiries can be directed to the corresponding authors.

## Supplementary Materials

The following supporting information can be downloaded at: https://www.mdpi.com/article/10.3390/ijms27104442/s1.

## Abbreviations

The following abbreviations are used in this manuscript:

CVD	cardiovascular disease
DEG	differentially expressed genes
IS	ischemic stroke
tMCAO	transient middle cerebral artery occlusion
